# Brain-wide associations of reaction time variability in the ABCD study

**DOI:** 10.1162/IMAG.a.18

**Published:** 2025-05-28

**Authors:** Thomas C. Maloney, Jonathan A. Dudley, Sarah L. Karalunas, Gowtham Atluri, John O. Simon, Leanne Tamm, Jeffery N. Epstein

**Affiliations:** College of Medicine, University of Cincinnati, Cincinnati, OH, United States; Department of Pediatrics, Cincinnati Children’s Hospital Medical Center, Cincinnati, OH, United States; Department of Psychology, Purdue University, West Lafayette, IN, United States; Department of Electrical Engineering and Computer Science, University of Cincinnati, Cincinnati, OH, United States

**Keywords:** variance time course, intra-individual variability, intra-subject variation in reaction time, attentional fluctuations, vigilance

## Abstract

Intra-individual variability in reaction times (IIVRT), which generally occurs as a result of episodic long reaction times (RTs), is a marker for impaired attention. Multiple functional neuroimaging studies have attempted to discern neurofunctional correlates of IIVRT, but few use models that account for trial-level IIVRT. Neurofunctional correlates of IIVRT differ depending on the method applied, and few studies have used multiple methods in the same sample. This study utilized Stop-Signal Task functional magnetic resonance imaging (fMRI) data from 8,066 children (9–10 years old) in the Adolescent Brain Cognitive Development (ABCD) study. IIVRT was modeled using multiple methods, including converting RTs to z-scores, variance time course modeling, and a novel machine-learning technique (i.e., hidden Markov model) to compute the probability of a trial reflecting good or poor attentional states. Across all three methods, lower IIVRT was associated with greater activation in the default mode network (DMN), while higher IIRVT was associated with greater activation in the dorsal attention network (DAN). Although all models yielded similar neural correlates, z-score modeling demonstrated the strongest effect sizes in task-related networks. Our findings are congruent with previous work in adults and demonstrate the reproducibility and developmental stability of the neural correlates of trial-level IIVRT. Higher effect sizes for brain-IIVRT associations using the z-score method suggest that this approach is a simple and promising candidate for investigating neural mechanisms related to IIVRT.

## 1 Introduction

Attention is crucial for sorting out relevant information and limiting the amount of information that gains access to further processing (Racer & Dishion, 2012). However, attention is not constant; it fluctuates considerably from moment to moment, likely due to multiple neurocognitive factors, including arousal, cognitive resource allocation, and effort (Esterman & Rothlein, 2019). Behavioral manifestations of this intra-individual variability in attention may include attentional lapses, mind-wandering, losing one’s train of thought, and so forth (MacDonald et al., 2009). Elevations in attentional variability are a transdiagnostic indicator of psychopathology, as evidenced by increased attentional variability in multiple disorders, including attention deficit hyperactivity disorder, autism, schizophrenia, and mood disorders, among others (Bora et al., 2006;Brotman et al., 2009;Castellanos et al., 2005;Epstein et al., 2011;Kaiser et al., 2008;Karalunas et al., 2014;Leth-Steensen et al., 2000).

Experimentally, intra-individual variability in attention is often measured by examining reaction time variability (IIVRT) on neurocognitive tests (Epstein et al., 2011). Moreover, these fluctuations in reaction time (RT) are assumed to correlate with neuronal activity, and several studies have attempted to discern the neurobiological basis of IIVRT. The majority of this research has examined associations between an overall scalar indicator of IIVRT (e.g., standard deviation of RT, coefficient of variation, ex-Gaussian tau) and regional brain activation (Bellgrove et al., 2004;Simmonds et al., 2007). These studies have generally found that higher IIVRT is associated with increased activation in the prefrontal cortex, parietal cortex, striatal, and thalamic regions (Bellgrove et al., 2004;Simmonds et al., 2007). Such associations between measures of IIVRT and brain activation are limited in that, depending on the indicator, they may a) reflect variability from both short and long RT trials; b) be highly correlated with RT speed; and c) may reflect a “third variable confound” driven by psychopathology’s associations with both IIVRT and altered neural response rather than direct relationships between neural response and individual RTs. Moreover, these RTV indicators (e.g., RTSD, tau) reflect average IIVRT across a whole task, whereas attention fluctuations actually occur episodically as moment-to-moment fluctuations in RT. Hence, more work is needed that directly examines trial-level associations between RTs and brain activation.

To date, a couple of methods have been utilized to understand trial-level neuronal activity in IIVRT (seeFig. 1). The variance time course (VTC) model assumes that individuals’ attention varies over time and that during the performance of a task, an individual will have periods where they exhibit reduced variation in RTs and periods where they exhibit elevated variation in RTs. The VTC model converts each RT in an individual’s RT time-series to*z*-scores based on that individual’s own mean and standard deviation and then takes the*absolute value*of those z-scores to capture how much the RT of a given trial varies from the within-person mean RT. Importantly, using this model, both very short and very long RTs become large, positive values. The implicit assumption is of a*non-linear*relationship in which increased variability (regardless of direction) corresponds to a state of reduced attention. While most studies have applied the VTC model to specific versions of a continuous performance test (i.e., gradual-onset continuous performance test) (Esterman et al., 2013;Esterman, Rosenberg, et al., 2014), subsequent research has also used the VTC model with go/no-go (Johnson et al., 2015), N-Back (M. D. Rosenberg et al., 2015), and finger tapping (Kucyi et al., 2017) tasks.

**Fig. 1.**
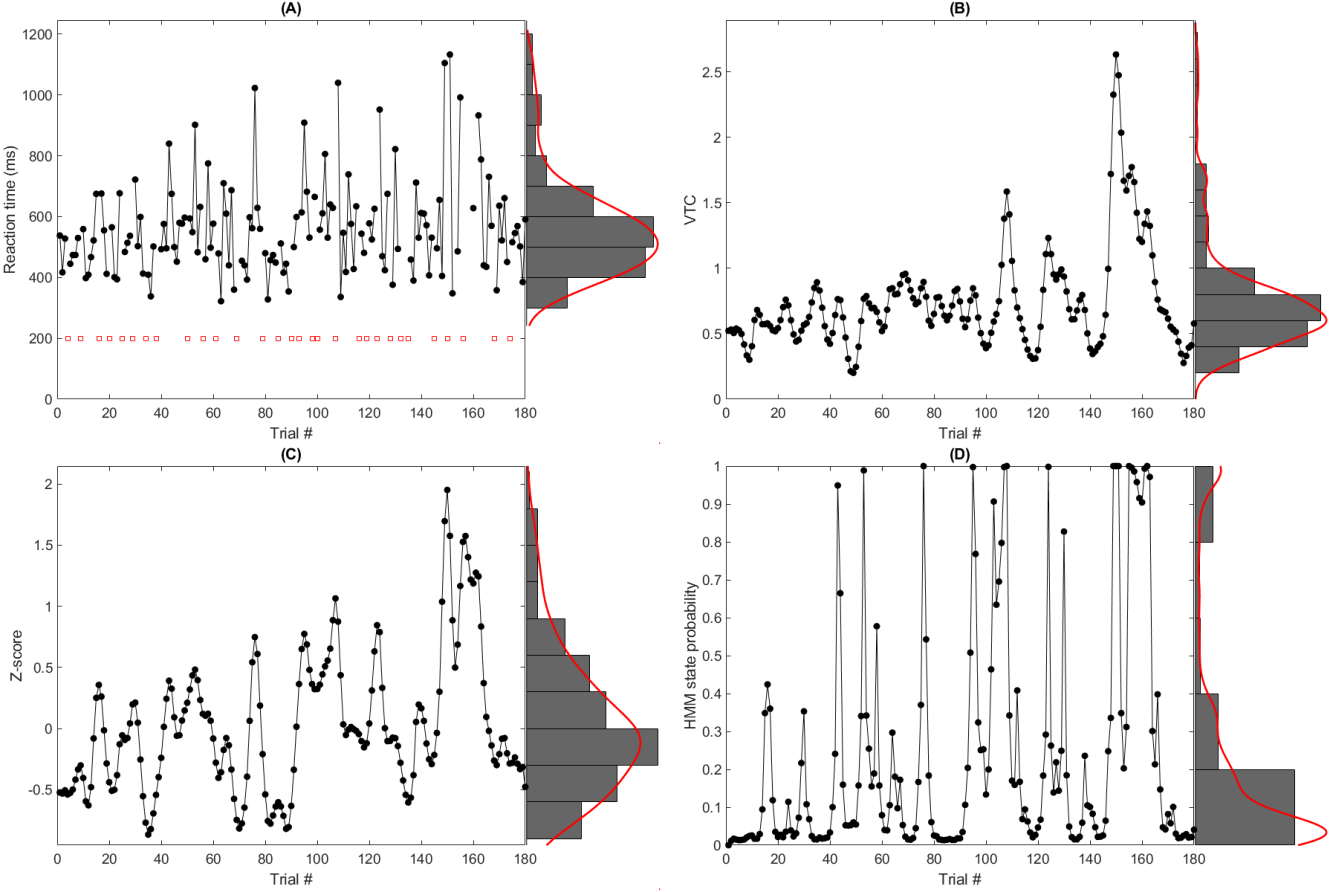
Example of one participant’s (A) trial-level reaction times and the three time-series transforms: (B) variance time course, (C) smoothed Z-score, and (D) hidden Markov model state probability. Red squares in tile (A) indicate stop trials.

The extant literature exploring the VTC model has largely focused on small samples (*n*< 50) of typically developing adults (ages 18 and above) (Esterman et al., 2013,^2015^,^2017^;Esterman, Reagan, et al., 2014;Esterman, Rosenberg, et al., 2014;Kucyi et al., 2017;M. Rosenberg et al., 2013;M. D. Rosenberg et al., 2015;Yamashita et al., 2021;Zuberer et al., 2021), with one exception (seeFortenbaugh et al., 2015which included a large sample ranging from 10 to 70 years of age but did not examine neuroimaging data). Behaviorally, results from these studies have been what one would expect. Individuals make more errors during periods of high IIVRT compared to periods of low IIVRT (Esterman et al., 2013;Esterman, Rosenberg, et al., 2014;Fortenbaugh et al., 2015,^2018^;Kucyi et al., 2016). Though results examining brain activation have been remarkably consistent, the pattern is somewhat counterintuitive: one might assume that better performance on a task would be achieved through stronger activation of brain regions involved with said task, and thus periods of low IIVRT would be associated with greater task-positive network activity, but the observed patterns are precisely the opposite. Specifically, at the trial-by-trial level, increased activity in task-positive (i.e., attentional and visual) networks and greater suppression of activity in task-negative (i.e., default mode network or DMN) networks predict more variable RTs (Esterman et al., 2013;Esterman, Rosenberg, et al., 2014;Fortenbaugh et al., 2018;Kucyi et al., 2016).Esterman et al. (2013)argued that being “out of the zone,” characterized by error-prone, variable performance, is cognitively challenging and that participants effortfully suppress DMN activity as they struggle to maintain task focus. In contrast, being “in the zone” may require less effortful control, which coincides with an increase in DMN activation. However, if the DMN activation becomes too high, this can predict subsequent task-related errors (Esterman et al., 2013;Esterman, Rosenberg, et al., 2014;Fortenbaugh et al., 2018).

Other investigators have examined*linear*associations between trial-level RT and brain activation (Grinband et al., 2011;Weissman & Carp, 2013a;Weissman et al., 2006;Yarkoni et al., 2009). This approach, which we will henceforth refer to as the z-score model, converts an individual’s RTs into within-person z-scores similar to the VTC but then examines associations between raw (rather than absolute value) z-scores and brain activation. Using the z-score model, high z-scores represent long RTs, whereas low z-scores represent short RTs. Differentiating trial-level activation during long versus short RT trials may be important for identifying specific brain activation patterns associated with potentially distinct cognitive processes of attentional lapses versus impulsive responses. Although the assumptions are different, methods analyzing a direct trial-by-trial relationship between z-scores and brain activation have yielded similar patterns of associations as the VTC approach, with long RTs being associated with increased activation in task-positive regions (Grinband et al., 2011;Weissman & Carp, 2013a;Weissman et al., 2006;Yarkoni et al., 2009) and decreased DMN activation (Barber et al., 2017) as compared to short RTs.

One limitation of the VTC and z-score models is that a few trials that significantly deviate (typically long RTs) from an individual’s mean RT can have a large effect on the results. For example,Yamashita et al. (2021)modeled gradCPT RTs using ex-Gaussian modeling and reported that brain states akin to “in the zone” (i.e., activation of DMN and limbic networks) and “out of the zone” (i.e., activation of frontoparietal, dorsal attention, somato-motor, and visual networks) did not differ on the exponential tau but did differ on sigma (variability of Gaussian) parameters. Hidden Markov models (HMMs) are a powerful class of machine-learning algorithms that assume an underlying process can be in one of ‘k’ states and data can be classified into ‘k’ distributions with unknown parameters. HMMs transition from one state to another using a state transition probability matrix. HMMs provide an effective approach to determining which data points are generated by each of the states, the parameters of the different distributions, and the state transition probability matrix. In our case, we apply HMM with two states to parallel the assumptions of the VTC and z-score models (i.e., in all three models, we assume some transform of RT is indicative of the individual’s attention spectrum from focused to reduced). The HMM overcomes potential limitations of the z-score model by modeling the underlying data using separate distributions for each state. It also allows for occasional trials with high or low RTs without changing from the previous state, as the probability of transitioning from one state to another is determined by a state transition probability matrix. Thus, this HMM approach will be less sensitive to noise in the individual RTs (e.g., a single slow RT due to, for example, a motor output problem while the person otherwise remains in a high attention state). Overall, HMMs allow for a data-driven, person-specific way to label attentional states based on the RT time-series that makes fewer distributional assumptions and are potentially less sensitive to trial-level noise.

To date, no studies have examined neural correlates of HMM states derived from trial-level RT data. Moreover, no study has applied both the VTC and z-score models with the same data or even using the same task. Thus, it remains unclear whether models yield similar information about trial-level neural correlates of IIVRT or whether models can provide unique information given their unique handling of aspects of the RT time-series. Further, nearly all studies have been done in college-age adults. And yet, there is considerable development in IIVRT that occurs in late childhood and adolescence as the brain regions responsible for attentional control mature, and developmental deviations in IIVRT may be associated with various forms of psychopathology (Boen et al., 2021;Thomson et al., 2020). Studies using trial-level approaches in younger samples are needed.

To address these gaps, this study utilized trial-level Stop Signal Task (SST) data from the ABCD sample to examine the neural correlates of all three models of IIVRT: a) VTC, b) z-score, and c) HMM. The Adolescent Brain Cognitive Development (ABCD) study (abcdstudy.org) sample provides an unprecedented opportunity to examine IIVRT in a large pediatric sample (*n*= 11,878 children). The sample includes children aged 9 to 10 years old at baseline, which helps attenuate the established impact of chronological age on IIVRT (Kofler et al., 2013). Yet the sample is heterogeneous in terms of sex, race, and psychological diagnoses (9% with a diagnosis on a semi-structured diagnostic interview with a caregiver), providing the necessary variance to study associations between IIVRT and behavioral and neurobiological outcomes. In this study, we explored the neurological correlates of VTC, z-score, and HMM computational estimates of RT to provide new insights into the development of IIVRT and its neural correlates during childhood.

## 2 Methods

### 2.1 Participants

The ABCD Study recruited 11,878 youth aged 9–10 years of age across 21 geographically diverse US sites (Compton et al., 2019). Informed consent was obtained from all participants prior to inclusion in the ABCD Study (Clark et al., 2018). The ABCD data is publicly available through the National Data Archive (NDA) with permission. In data release 4.0, 11,402 children had baseline imaging data, of which 9,691 participants had at least one run of the Stop Signal Task that passed the ABCD study’s quality control. We excluded children whose accuracy on the SST was <66% on Go trials (n = 1089) or whose mean stop probability was <25% or >75% (n = 157; 61 of whom had accuracy >66%). Eleven children were removed due to non-convergence of the HMM. Hence, the sample size for the current study was 8,055 children (mean age = 9.9,*SD*= 0.63; 49.2% female; 19.8% Hispanic, 77.5% White, 17.9% Black/African American, 3.4% American Indian, 0.2% Native Alaskan/Hawaiian, 0.5% Pacific Islander, 6.6% Asian, 6.2% other, 0.4% refused to answer, 0.9% do not know).

### 2.2 Behavioral paradigm

The SST is a computerized measure of response inhibition with 2 runs of 180 trials each (Casey et al., 2018). On every trial, the participant views a horizontal arrow pointing either right or left. Participants indicate the direction of the arrow via a two-button response panel within 1,000 ms after which a fixation cross appears with a jittered inter-trial-interval that lasts from 700 to 2,000 ms. Thirty (16.6%) trials in each run were Stop trials on which the horizontal arrow is followed by an upward arrow (i.e., the stop signal) for 300 ms. Participants are directed to inhibit their response when they see the stop signal. The delay between presentation of the horizontal target arrow and the upward arrow (stop signal delay; SSD) begins at 50 ms and varies according to the participant’s performance. Successful inhibition results in increases of 50 ms, and unsuccessful inhibition results in decreases of 50 ms so that the rate of inhibition is approximately 50%. Task accuracy was computed as the ratio of correct go-trial responses to the total number of go-trials.

### 2.3 Data processing

#### 2.3.1 RT time-series

All approaches require a string of RTs for all trials. We included all available RTs from Go trials where RT >150 ms, including incorrect Go trials (8%) and RTs that occurred after the 1,000 ms response period (i.e., during the ISI; 0.3%). Finally, consistent with the VTC approach, to estimate RTs for all Stop trials, regardless of accuracy, and Go trial omissions (22.7% of trials; seeFig. 2for proportion of different trial types), linear interpolation using the*z*-scores of the nearest trials before and after the target trial was used (Esterman et al., 2013;Esterman, Rosenberg, et al., 2014;M. Rosenberg et al., 2013).

**Fig. 2.**
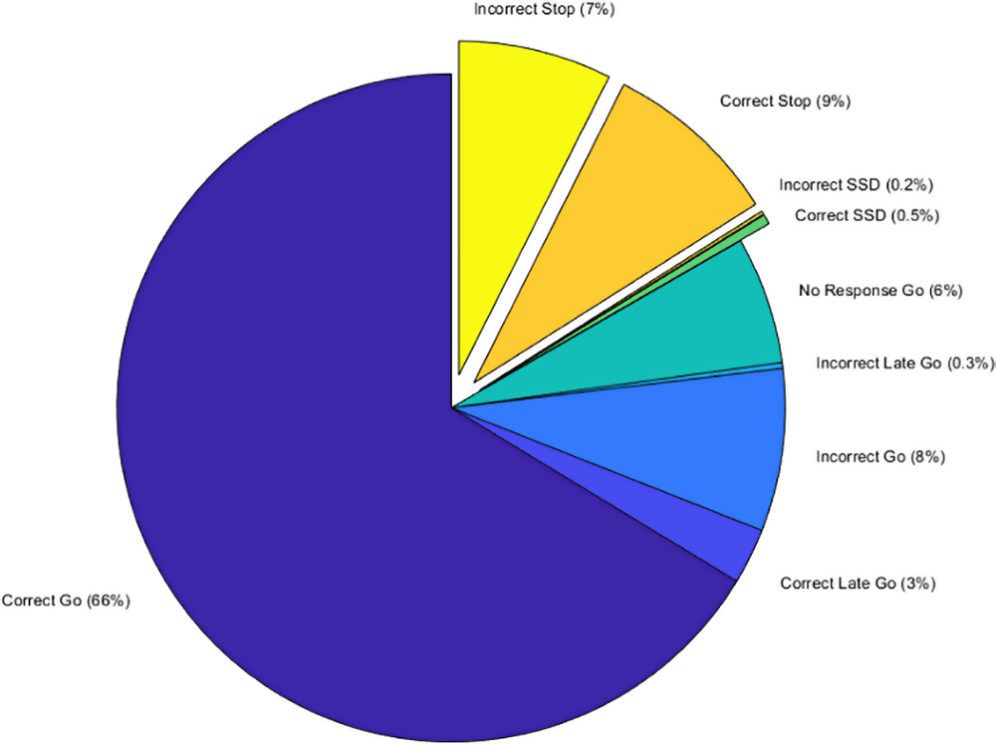
Distribution of stop signal task trial types.

#### 2.3.2 VTC estimation

To estimate the VTC, RTs were converted to absolute*z*-scores using the within-subject mean and SD of RTs for each run. Each time course was then subjected to Gaussian smoothing using a 7.2 s full width, half maximum (FWHM). These trial-level VTC weights were used for neuroimaging analyses.

#### 2.3.3 z-score estimation

Similar to the VTC computation, RTs were converted to z-scores using the within-subject mean and SD of RTs for each run. Each time course was then subjected to Gaussian smoothing using a 7.2 s full width, half maximum (FWHM). These scores were used for neuroimaging analyses.

#### 2.3.4 HMM estimation

Gaussian HMMs (Zucchini et al., 2016) are generative models that assume that the system being investigated will be in one of the multiple possible states and the observations (also referred to as*emissions*) from the system follow a Gaussian distribution. We modeled each subject’s RT time course separately using Gaussian HMMs, with the assumption that, at each trial, the participant’s attentional state is either focused or not and that the RTs ‘emitted’ from each of these states follow a Gaussian distribution. Specifically, the Baum-Welch algorithm is used to learn the mean and variance of the Gaussian distribution of the RTs for each of the two states and a transition probability matrix that determines the probability of transitioning from one state to another (Rabiner, 1989). A Viterbi approach is used to estimate which state the subject is in at any instance (Lou, 1995). We used the implementation of these two algorithms from the Gaussian HMM function available in the Python hmmlearn package (hmmlearn). The model returns a probability at each time point between 0 (state A) and 1 (state B); because these states do not necessarily correspond across subjects, we set as state B (probability score of 1) whichever state had longer mean RTs by subtracting the model output from 1, if necessary. In this manner, higher HMM model values positively correlate with reaction time, similar to the z-score model. The resulting vector of state probabilities was used as the continuous time-series variable in comparison with the VTC and z-score models.

### 2.4 Neuroimaging

#### 2.4.1 Image processing

Baseline SST fMRI and minimally processed T1 imaging data from the ABCD study, release 4.0, that passed the ABCD study’s quality control were analyzed. The fMRI was acquired with the following parameters: 60 slices, resolution = 2.4 × 2.4 × 2.4, TR = 800 ms, TE = 30 ms, flip angle = 52°, FOV = 216 × 216 mm, and multiband acceleration = 6. T1 scan parameters were: 176 slices, resolution = 1.0 × 1.0 x 1.0, TR = 2,500 ms, TE = 2.88 ms, flip angle = 8°, and FOV = 256 × 256 mm. Scanning parameters were harmonized across sites (Casey et al., 2018). After downloading, T1 images were skull stripped using FSL’s brain extraction algorithm*bet*(Smith, 2002). Brain images were normalized to 1 mm isotropic MNI space (Grabner et al., 2006) using the linear registration implemented in FSL’s*flirt*(Jenkinson & Smith, 2001;Jenkinson et al., 2002). Images were then segmented into white matter (WM), grey matter (GM), and cerebrospinal fluid (CSF) regions using FSL’s*fast*(Zhang et al., 2001) and subcortical regions using FSL’s*first*(Patenaude et al., 2011).

Minimally processed fMRI data was used to compute the measures of interest for the SST for each of the three RT models. We performed additional pre-processing in accordance with the ABCD pre-analysis processing steps (Hagler et al., 2019), that is, removal of initial frames and normalization of voxel time-series.

### 2.5 Statistical analyses

#### 2.5.1 fMRI regression analysis

Voxelwise association maps for each of the RT time-series models were generated for each subject using a two-step regression procedure wherein signal variance associated with nuisance regressors (21 parameter model that included the x, y, and z components of translation and rotation along with their first derivatives and squares, and the mean signal within the WM, CSF, and whole brain) was first removed via ordinary least-square regression. Next, RT time-series effects were estimated for each RT time-series model separately by performing an amplitude-modulated linear regression fit of these residuals to the given model time-series convolved with a Gaussian hemodynamic-response function. Activation from multiple runs of the SST task was averaged at the subject level using a fixed-effects analysis in FSL’s*flameo*(Woolrich et al., 2001). Voxelwise group activation was calculated using FSL’s*fsl_glm*(Jenkinson et al., 2012).

#### 2.5.2 Post hoc analysis

We additionally modeled the trial-level activation for each participant to examine the relationship between task performance and relative activation in brain regions found to be either positively or negatively correlated with each RT time-series. For this analysis, trial-level design matrices were generated with one regressor for each trial (regardless of trial type); the onset and duration for each regressor were taken as the go-stimulus onset time and RT, respectively, of the corresponding trial. Regressors were convolved with a canonical hemodynamic response function. FSL’s*fsl_glm*was used to model subject-level effects, generating a whole-brain map of parameter estimates for each trial for each participant. Trial-level activation data were used to estimate the effect size of each RT time-series metric on BOLD activation. First, BOLD data were aggregated into the Gordon functional atlas, delineating the brain into 333 regions of interest, each categorized into one of 13 functional networks (Gordon et al., 2016). Then, for each participant, the Pearson correlation coefficient was computed between their go-trial BOLD signal parameter estimates and their RT time-series. Cohen’s*d*effect size was then estimated from a linear regression model with centered age and sex included as continuous and categorical covariates.

To compare the brain-wide associations of the three models, the Dice score was computed as a measure of the overlap of the activation maps. Additionally, we computed the pairwise correlations of each activation map. Statistics from the correlation were determined using 10,000 permutations of the spatial null models (Alexander-Bloch et al., 2018), as implemented by the Neuromaps Python package (Markello et al., 2022).

## 3 Results

### 3.1 Performance distribution

The relationship between accuracy and RT time-series was plotted to better understand how accuracy was associated with RT scores in each model (seeFig. 3). In regard to raw RTs (untransformed), as would be expected, most go-trial errors were made with very fast RTs (<450 ms) or very long RTs (>1,200 ms). Consistent with what was observed for raw RTs, the Z-scores displayed a parabolic relationship where performance is lowest for very negative and very positive z-scores (i.e., very short and very long relative RTs) and highest for z-scores closer to zero. With VTC estimates, there was a monotonically negative relationship with performance where high VTC scores (i.e., very short and very long relative RTs) were characterized by the lowest relative accuracy and low VTC scores (i.e., RTs closest to the participant’s median RT) were characterized by the highest relative accuracy, again as would be expected and consistent with the pattern observed for the other RT models. Finally, HMM exhibited less pronounced differences in accuracy across the time-series, with only slightly lower accuracy observed for RTs with very low probability of being in the “longer RT” state (i.e., lowest accuracy for shorter RTs).

**Fig. 3.**
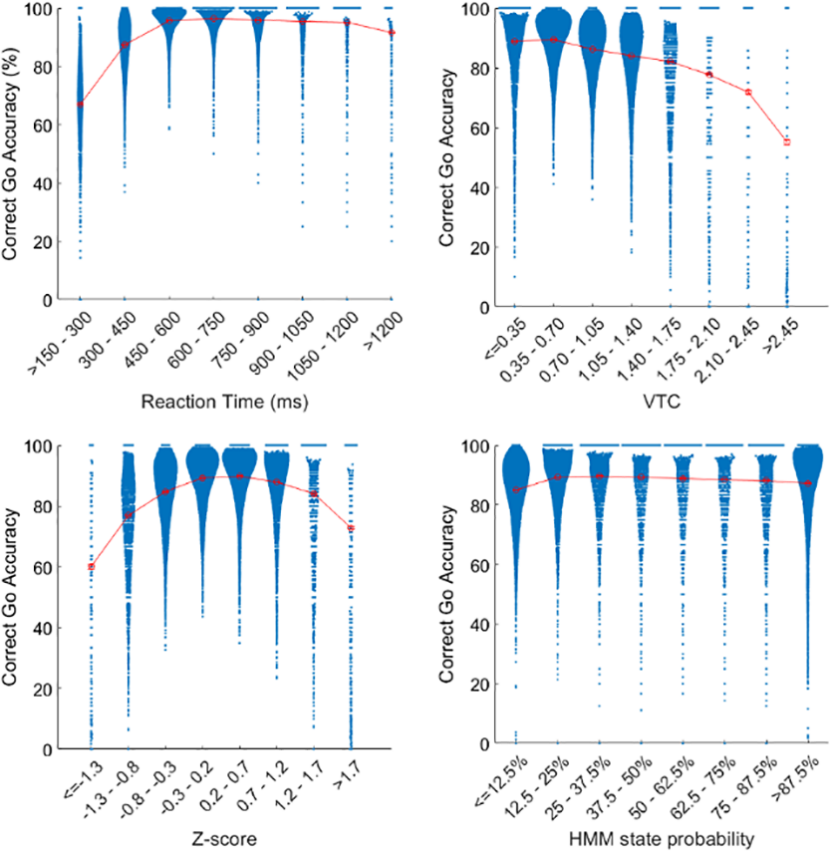
Violin plots showing the change in performance with change in RT time-series. The dots in each violin represents an individual participant where the y-value is the correct-go accuracy that participant achieved across all go trials for which their RT time-series values fell within the range indicated on the x-axis. In red are the mean and standard error of the achieved correct-go accuracy for each bin.

### 3.2 Regression analyses

All three IIVRT models showed similar patterns of brain-wide associations. As individuals’ IIVRT model values increased (higher z-scores, higher VTC scores, higher probability of being in a “long RT” state), there were negatively correlated regions in DMN areas and positively correlated regions in cingulo-opercular, somatomotor, and dorsal attention network areas (seeFig. 4).

**Fig. 4.**
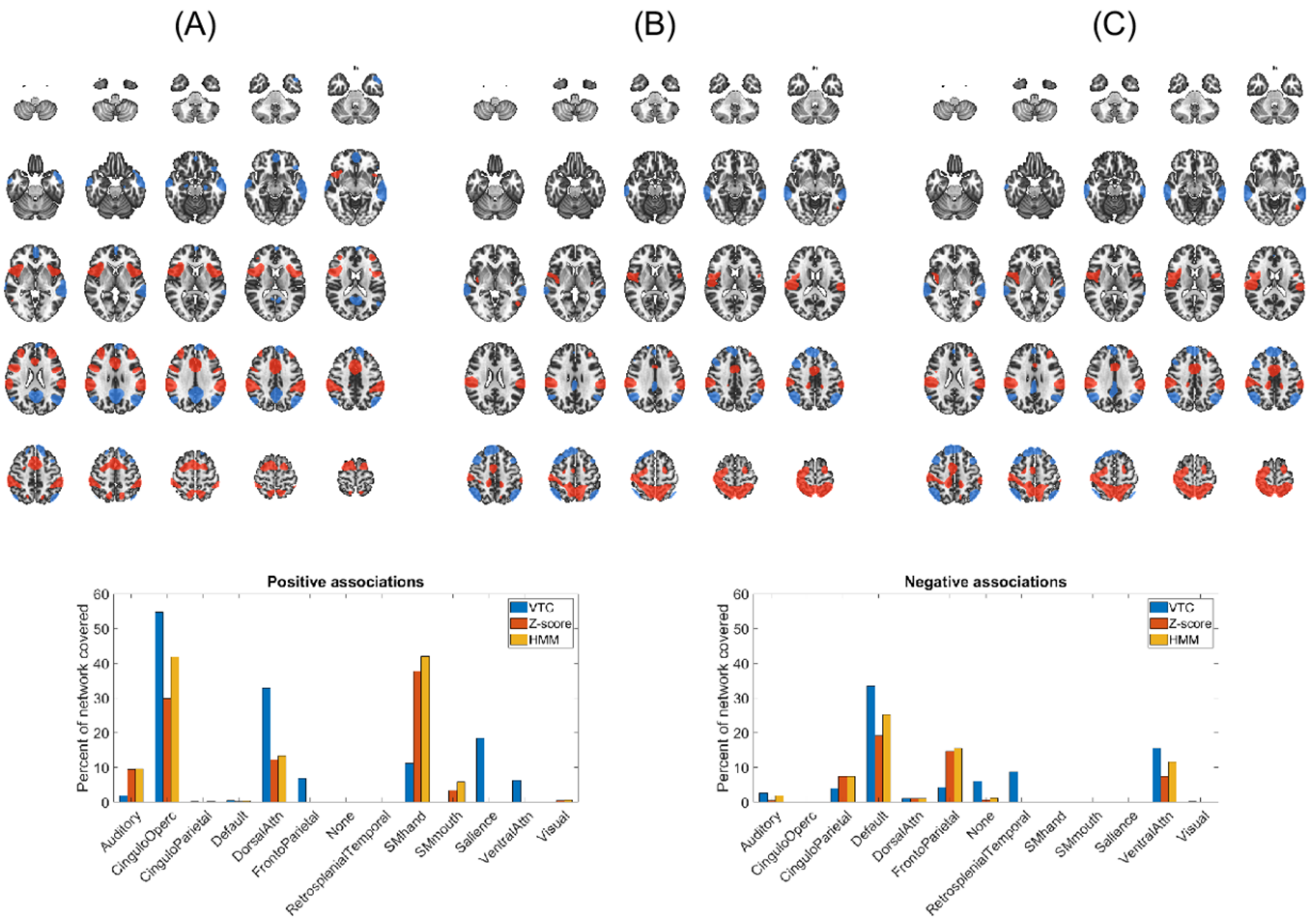
Group brain-wide association images at a z > 14 threshold for the RTV models (A) VTC, (B) z-score, and (C) HMM. Areas that are positively correlated with the RTV models are shown in red-yellow and negatively correlated areas in blue-light-blue. Below, bar plots at left and right show the percent of each atlas-defined network that is covered by the suprathreshold positive and negative clusters, respectively, for each model.

While all three methods had similar patterns of activation, the z-score and HMM models yielded the most similar patterns of brain-wide associations to one another (dice score of positive clusters: 0.88; dice score of negative clusters: 0.85); comparatively, the patterns of the VTC and z-score models (positive: 0.32; negative: 0.27) and VTC and HMM models (positive: 0.39; negative: 0.37) exhibited considerably less overlap. The z-score and HMM models showed greater positive associations, compared with the VTC model, in regions of bilateral superior parietal, bilateral post central, right middle cingulum, and bilateral regions that extended into the rolandic operculum. In contrast, the VTC model showed greater positive associations in the regions of bilateral inferior operculum and bilateral insula, bilateral middle frontal, and cingulum. For task-negative regions, the z-score and HMM models showed greater negative associations in the regions of the right superior medial frontal, bilateral middle frontal, and right middle temporal. The VTC showed greater negative associations in the regions of left middle temporal, bilateral hippocampus, left frontal inferior orbital, bilateral frontal medial orbital, and bilateral precuneus.

**Fig. 5.**
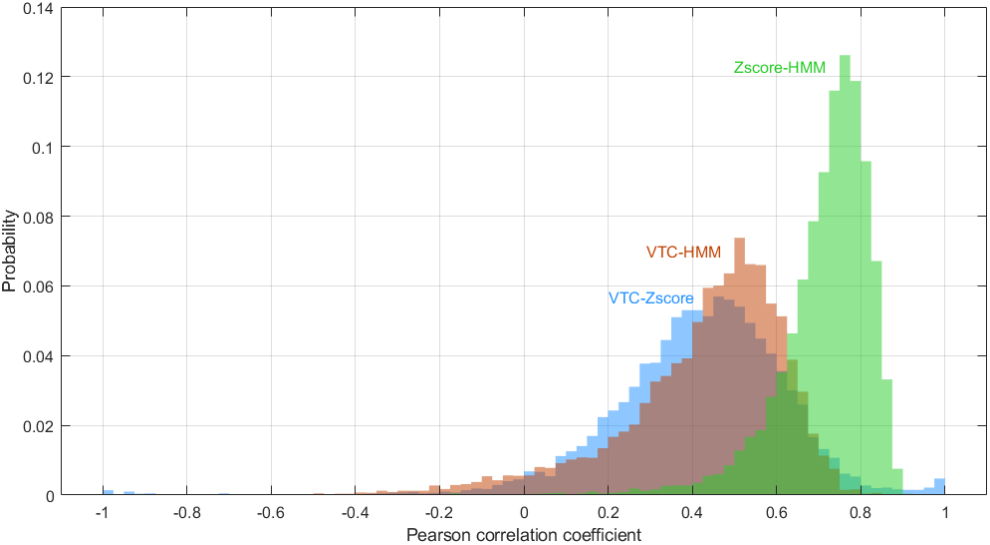
Histograms of the within-subject Pearson correlations of the three RT time-series models. The correlations are shown between the VTC and Z-score (blue), VTC and HMM (brown), and the Z-Score and HMM (green).

The similarities and differences in neural correlates across the three methods are perhaps not surprising, given that the z-score model and HMM estimates are the most similar on average, while the VTC and z-score models are the least similar on average.Figure 5shows the histograms of the within-subject Pearson correlations of the three RT time-series models. We computed the pairwise correlations, using spatial null models, of the three RT group activation maps and found all are strongly correlated with the z-score and HMM models having the strongest correlation (VTC to z-score: r = 0.69, p < 0.0001; VTC to HMM: r = 0.74, p < 0.0001; z-score to HMM: r = 0.99, p < 0.0001).

### 3.3 Post hoc analysis

**Fig. 6.**
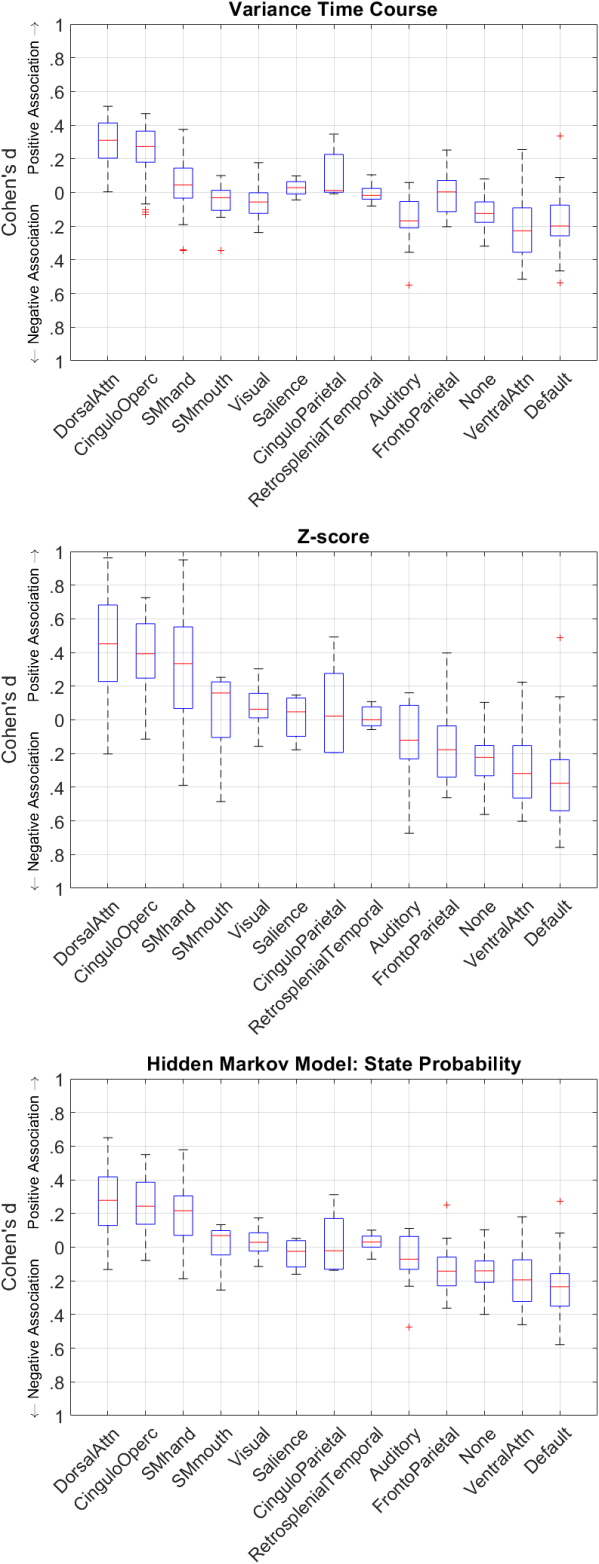
Cohen’s*d*effect sizes of VTC (top), Z-score (middle), and HMM (bottom) on BOLD activation during go-trials. The sign of effect size (positive/negative) indicates whether the RT time-series-BOLD association during go-trials was, on average, positive or negative. Each box-whisker plot shows the median, interquartile range, and extrema of effect sizes for each ROI in the given functional Gordon atlas networks. Network order is sorted from strongest positive to strongest negative median effect size of the Z-score RT time-series.

Figure 6shows the effect sizes for each of the IIVRT model values on BOLD activation by the functional network. All three time-series exhibited the strongest positive associations with BOLD activation in regions within dorsal attention, cingulo-opercular, and somatomotor hand networks. All three time-series exhibited the strongest negative associations in regions within the ventral attention network and DMN. Comparatively, the z-score time-series exhibited the strongest effect sizes in the task-related networks.

## 4 Discussion

In this work, we describe three approaches to modeling trial-level RT variability and characterize the BOLD correlates of each model for a very large sample of early adolescents performing a Stop Signal Task. Two of these models—the VTC and z-score—have been previously employed by other investigators but, to our knowledge, have rarely been utilized in a pediatric population and certainly not in a sample of this size. Further, this is the first work directly comparing these models in the same sample. We also present a novel model of trial-level RT variability in the HMM approach. Broadly, findings indicate that decreased DMN and increased DAN are associated with greater IIVRT on a trial-by-trial basis. Our results are, by and large, congruent with these previous works and thus demonstrate the reproducibility and generalizability of studies investigating the neural basis of IIVRT, as well as the developmental stability of the neural correlates of IIVRT.

### 4.1 Task performance as a function of model time-series

The relationship between each model and task accuracy was examined to better understand the impact of the various transformations of trial-level raw RTs into trial-level indicators of RT performance. Based on raw RTs, the majority of incorrect responses tended to occur during short RT trials (Fig. 3, top left panel), likely representing a speed-accuracy tradeoff. Importantly, very long RTs were*also*associated with lower accuracy, potentially representing attentional lapses due to mind wandering, mind blanking, or increased sensitivity to external distractions (Unsworth & Miller, 2021). Because the VTC model takes the absolute value of z-scored RTs, thereby weighting all fast and slow RTs with higher VTC scores, it is not surprising to see a monotonically negative relationship between accuracy and VTC score (Fig. 3, top right panel). In this manner, the VTC model provides a useful scalar value at the trial level wherein higher values are more likely to reflect either impulsive responding (i.e., fast RTs) or attentional lapses (i.e., slow RTs), both of which represent sub-optimal attentional states. The z-score model, on the other hand, has a parabolic relationship with accuracy (Fig. 3, bottom left panel), as the lowest*and*highest scores had the highest tendencies toward incorrect responses. Thus, the z-score model can be considered to provide a scalar value at the trial level that reflects the participants’ attentional state on a spectrum from impulsive to inattentive. Thus, both approaches provide useful information and the selection of approach for any given question would rely on whether the goal was to differentiate impulsive and inattentive responding or to address questions generally about good versus poor performance states.

Given the parabolic relationship between z-scores and accuracy, it is perhaps not surprising that the HMM does not exhibit a strong performance discrimination (Fig. 3, lower right panel). The model’s sole assumption was that there are two states defined by RT only. A three-state HMM may exhibit better performance discrimination as it could assign each trial a probability of being in an impulsive, inattentive, or optimal performant state. However, for the goals of the current study, which focused specifically on trial-level RT variation, a two-state model is a more appropriate choice to examine neural correlates of IIVRT states. Like the VTC and z-score models, which consider trial-level RT variation as a continuous vector along a single dimension (as low- to high-variance and fast to slow reaction times, respectively), the two-state HMM presumes a fast and slow RT state and assigns each trial a probability of where it falls along that dimension. Future studies could consider a three-state model, which would require simultaneously considering three separate probability time-series (fast medium RT, medium slow RT, and fast slow RT), each yielding its own performance curve and neural correlate map with their own distinct interpretations.

### 4.2 BOLD correlates of model time-series

Efforts to explore the neural systems involved in IIVRT have suggested critical roles for the dorsal frontoparietal attention network (DAN). The DAN is engaged by goal-directed attention and closely interacts with other networks involved in task performance, such as the cingulo-opercular, ventral attention, and salience networks. Regions spanning these networks have consistently been shown to exhibit positive associations with RTs during various tasks involving a diverse array of cognitive processes (Grinband et al., 2011;Mumford et al., 2024;Weissman & Carp, 2013b;Yarkoni et al., 2009). As shown inFigure 5, all three of our models also exhibit positive associations with regions in these task-positive networks. Previous studies have provided evidence that positive associations between RT and BOLD activity in these regions are due to “stimulus processing time”—that is to say, the higher observed BOLD signal amplitude for longer RT trials is explained by longer durations of neuronal activity during long RT trials rather than stronger amplitudes of neuronal activity (Grinband et al., 2011;Yarkoni et al., 2009). However, the VTC model also exhibits positive associations with BOLD activity in task-positive networks both in our study (Fig. 4A) and in previous work (Esterman et al., 2013;Esterman, Rosenberg, et al., 2014;Fortenbaugh et al., 2018;Kucyi et al., 2016) despite the fact that very short RTs, as well as long RTs, comprise high VTC scores.

In contrast to task-positive networks, the DMN is a network of brain regions that includes the precuneus/posterior cingulate cortex (PCC), medial prefrontal cortex (MPFC), and medial, lateral, and inferior parietal cortex that is generally deactivated during acts of attentional control with greater activity during rest than task performance (Raichle, 2015). Associations with RT in DMN regions have been less studied than their task-positive counterparts. Barber et al. observed longer RTs to be associated with increased suppression of DMN regions during a “simple” Go/No-go task (which is comparable to the Stop Signal Task in this study), congruent with our observations in the z-score model (Fig. 4B). Interestingly, Barber et al. observed a de-coupling of this relationship occurred during a more cognitively demanding task involving working memory (Barber et al., 2017). Again, the VTC model similarly exhibits greater suppression of activity in the DMN with increasing scores (i.e., long*and*short RTs), both in our study (Fig. 4A) and in previous work (Esterman et al., 2013;Esterman, Rosenberg, et al., 2014). That suppression of the DMN can occur during either periods of inattentiveness (long RTs) or periods of high RT variability (high VTC scores) suggests that the DMN is dynamically suppressed when task performance declines along a behaviorally relevant dimension (i.e., speed and accuracy).

Because this is the first study to employ these different models of IIRTV in the same dataset, it is of interest to explore our observed differences between the VTC, z-score, and HMM maps. Notably, we observed positive VTC associations to span more of the cingulo-opercular, and dorsal attention, networks compared to z-score and HMM (Fig. 4, bottom left). Importantly, the VTC has positive associations in the salience network that are not present in the z-score or HMM maps—in the VTC map, the areas of the salience network are bilateral insular regions (Fig. 4A) which are highly involved in performance monitoring/error detection (Dosenbach et al., 2006;Seeley, 2019). We hypothesize that these associations are driven by very short RTs (which have high VTC scores) that are more likely to represent an impulsive and error-prone state and are thus associated with greater activation in salience network regions (Ham et al., 2013). Conversely, the z-score and HMM-positive association maps span much more of the somatomotor hand network (i.e., medial portions of the precentral and postcentral gyri and sulci) compared to the VTC map. This could perhaps be explained by the same “stimulus processing time” effect cited earlier. The longer it takes a person to respond on a trial, the longer the hand somatomotor network is engaged. Since high VTC scores also include short RT trials, we might expect less activation in hand somatomotor regions compared to z-score and HMM scores. Overall, the three models of trial-level RTV exhibit similar patterns of brain-wide associations. Despite these similarities, the z-score model shows the strongest effect sizes in task-related networks (Fig. 6) and may thus be a more promising candidate for investigating neural mechanisms related to IIRTV. It may be that the VTC model may reduce “stimulus processing time” effects, and that the reduced effect sizes may be a marker of greater specificity.

### 4.3 Limitations

While this study used a large, diverse sample and a variety of methods to examine the neurobiological basis of RTV, there are some limitations. First, each approach to defining deviant RTs involves assumptions that may not be accurate for all participants. The assumption of two states by the HMM was discussed above. In addition, both the VTC and z-score models assume that all participants are equally variable in their RTs because scores are created relative to a person’s own RT time-series. As a result, a similar z-score might have different interpretation in participants with low RTV and high RTV; a low IIVRT participant may have trials assigned high z-scores even though the raw RTs on that trial are not abnormally long relative to the distribution of RTs recorded across all participants. This may attenuate the ability to find neurobiological associations between VTC/z-score weightings and BOLD response if different mechanisms drive RT variability depending on how much variation is present. However, if similar neural mechanisms are responsible for within-person variation in RT, regardless of the degree of variability, then this would not be a problem.

Second, the majority of the literature examining neurobiological associations with RTs has utilized cognitive tasks that require a response on all trials. Because the ABCD study used the SST which has stop trials where RTs are not relevant, we were forced to make assumptions regarding trials without RTs. Specifically, interpolation was used to account for stop trials (16.6% of trials) as well as other trials where an RT was not recorded (e.g., omission errors). While interpolation is also commonly used in VTC and z-score approaches for omission trials, the use of the SST required interpolation on many more trials than in previous research.

Further, measuring RT variability in the context of a stop task may have affected both the cognitive processes activated on go-trials and the neural correlates. Although the go and stop processes on tracking versions of the stop signal task are considered to be largely independent, participants do sometimes make proactive adjustments to response strategy on go trials (i.e., slowing go RT to increase likelihood of success on stop trials). These proactive control adjustments are generally small and are minimized further by tracking procedures and low proportions of stop trials as was done on the current task (Verbruggen & Logan, 2009). In addition, recent studies have found distinct neural correlates for stop response accuracy and RTV, suggesting unique rather than largely overlapping mechanisms (Lee & Kang, 2020) and attentional deficits rather than differences in proactive control may actually primarily drive longer SSRTs (Weigard et al., 2019). Finally, prior work suggests that children in this age range are less likely to use proactive control adjustments than their older adolescent peers. Nonetheless, the specific task use may have attenuated or confounded the RT-BOLD associations reported here, and we can also not definitively say whether DAN-RT relationships reflect differences in reactive or proactive control strategies. Additional studies using tasks with different cognitive demands are needed.

Finally, while the ABCD sample is unprecedented in terms of its size and geographic, gender, racial, ethnic, and diagnostic diversity, it was beyond the scope of this manuscript to examine whether group differences in the neurobiological bases of RTV may differ across any of these sociodemographic and clinical groupings.

## 5 Conclusions

Despite these limitations, these findings add to the literature by replicating and expanding our understanding of the neural basis of attentional variability. Across all three methods, lower IIVRT was associated with greater activation in the DMN, while higher IIRVT was associated with greater activation in the DAN. Our findings demonstrate the reproducibility and developmental stability of the neural correlates of trial-level IIVRT in children. Higher effect sizes for brain-IIVRT associations using the z-score method suggest that this approach is a promising candidate for investigating neural mechanisms related to IIRTV. Future studies should explore whether effects replicate across independent subsamples of participants.

## Data Availability

Data from the ABCD study is available from the NDA (https://nda.nih.gov). Code for generating the VTC, z-score and HMM models is available from the authors upon request.
